# *In Vitro* Anthelmintic Activities of *Khaya anthotheca* and *Faidherbia albida* Extracts Used in Chad by Traditional Healers for the Treatment of Helminthiasis and *In Silico* Study of Phytoconstituents

**DOI:** 10.1155/2024/8564163

**Published:** 2024-06-28

**Authors:** Baigomen Christalin, Masoud Besati, Noumedem Anangmo Christelle Nadia, Mahdi Yaghoobi, Yamssi Cédric, Claire Ciancia, Mounvera Abdel Azizi, Gamago Nkadeu Guy-Armand, Vincent Khan Payne, Haibo Hu

**Affiliations:** ^1^ Department of Animal Biology Faculty of Science University of Dschang, P.O. Box 067, Dschang, Cameroon; ^2^ Laboratory of Tropical and Emerging Infectious Diseases, Dschang, Cameroon; ^3^ Institute for Integrative Systems Biology (I2SysBio) CSIC-University of Valencia, Paterna 46980, Spain; ^4^ Department of Microbiology Haematology and Immunology Faculty of Medicine and Pharmaceutical Sciences University of Dschang, P.O. Box 96, Dschang, Cameroon; ^5^ Molecular Design and Synthesis Department of Chemistry KU Leuven, Celestijnenlaan 200F, Leuven B-3001, Belgium; ^6^ Department of Biomedical Sciences Faculty of Health Sciences University of Bamenda, P.O. Box 39, Bambili, Cameroon; ^7^ Wellcome Centre for Molecular Parasitology School for Infection and Immunity University of Glasgow, Glasgow, UK; ^8^ National Engineering Research Center for Modernization of Traditional Chinese Medicine-Hakka Medical Resources Branch School of Pharmacy Gannan Medical University, Ganzhou, China

## Abstract

**Background:**

Helminthiasis is endemic in Chad and constitutes a public health problem, particularly among school-age children. The aim of this study was to evaluate the anthelmintic activity of extracts of *Khaya anthotheca* and *Faidherbia albida* used in Chad by traditional healers for the treatment of helminthiasis.

**Methods:**

The anthelmintic activity was assessed against *Heligmosomoides polygyrus* and *Caenorhabditis elegans* larvae using the Worm Microtracker. Embryonated eggs, L1, L2, and L3 larvae of *H. polygyrus* were obtained after 24 h, 48 h, and 7 days of coproculture and L4 larvae of *C. elegans* culture using standard procedures. One hundred microliters of extracts at various concentrations, with albendazole and distilled water were, put in contact with 100 *µ*L of *H. polygyrus* suspension (containing 50 parasites at various developmental stages) in a microplate and incubated for 20 h at 25°C in the Worm Microtracker. The same procedure was adopted for *C. elegans*, but with 180 *µ*L of OP50. 19 *µ*L of *C. elegans* suspension (containing 50 larvae) was put in contact with 1 *µ*L of extract at various concentrations and incubated in the Worm Microtracker. Docking studies were carried out using the Schrodinger Maestro software's Glide module. The score function in the software was used to rank and group distinct possible adduct structures generated by molecular docking.

**Results:**

The aqueous and ethanolic extracts of *F. albida* at a concentration of 2.5 mg/mL showed the same activity as albendazole (100 ± 0.00) on hatching. The IC50s of the aqueous extracts of the two plants (IC_50_: 0.6212 mg/mL and 0.71 mg/mL, respectively) were comparable on egg hatching of *H. polygyrus* with no significant difference (*p* ≥ 0.05) with respect to the ethanol extracts (IC_50_: 0.70 mg/mL and 0.81 mg/mL, respectively). There was no significant difference between the percentage inhibition of extracts and albendazole on the L1 larvae of *H. polygyrus* (*p* ≥ 0.05). The aqueous extracts acted more effectively than the ethanol extracts on the L1 larvae of *H. polygyrus* with an IC_50_ of 0.5588 and ∼9.858*e* − 005 mg/ml, respectively, for *K. anthotheca* and *F. albida*. The aqueous extracts of *K. anthotheca* and *F. albida* on L3 larvae of *H. polygyrus* had inhibitory percentages of 92.6 ± 0.62 and 91.37 ± 0.8 at 2.5 mg/mL which were lower than albendazole (100 ± 0.00). The aqueous extracts of *K. anthotheca* and *F. albida* on *C. elegance* showed IC_50_ of 0.2775 *µ*g/mL and 0.5115 *µ*g/mL, respectively, and were more effective than the ethanol extracts. Examining *K. anthotheca* and *F. albida* through the interaction with the protein receptor and its results also confirmed our assumption that the compound used has hydroxyl and carbonyl groups as well as aromatic rings and is exposed to phenolic and flavonoid groups in a more specific way, and it shows a better inhibitory effect.

**Conclusions:**

This study scientifically validates the use of extracts of the two plants in the traditional treatment of helminthiasis. However, it will be necessary to evaluate the *in vivo* anthelmintic activity and toxicity. Examining the ADME properties of these compounds also supports the potential of these ligands to be transformed into pharmaceutical forms.

## 1. Introduction

The poorest and most neglected communities are among those infected by soil-transmitted helminth infections, which are the most prevalent infections worldwide [1]. Different species of parasitic worms are the cause of helminth infections that are spread through the soil [2]. They are spread by eggs found in human feces, which contaminate the soil especially when sanitary conditions are improper. Children with infection have nutritional and physical deficiencies. Periodic deworming to remove infected worms, health education to avoid reinfection, and improved sanitation to lower soil contamination with infectious eggs are the main components of control [3]. Because the disease is neglected in most African countries, little effort is being made to eradicate these diseases. A study conducted by Samafou et al. [4] on intestinal helminthiases among school children in the Sahelian and Sudanian zones of Chad revealed an overall infection rate of 35.87% in both zones, with the most frequent helminths being *Ascaris lumbricoides* (16.41%), *Schistosoma mansoni* (14.0%), and *Hymenolepis nana* (6.53%). Brooker et al. [5] estimated that 32.7% of all rural Chadians are affected with ankylostomiasis. In the city of N'Djamena, Kostoingue et al. [6] observed that 57.7% of kids have intestinal parasites. According to recent research, 51% of people living in N'Djamena city are carriers of at least one intestinal parasite, and 60% of Chadian children who live in nomadic communities [7]. All these high prevalences of helminths lead to one conclusion that this disease is seriously neglected in Chad.

The frequent use of synthetic anthelmintics has resulted in nematode resistance to existing synthetic medications, which has negative effects such as limited efficacy. In addition, the lack of effective anthelmintics, inadequate quality, and the relatively expensive cost of medications prevent some families from getting access to them and receiving regular treatment [8]. This is why there is a pressing need to identify and develop novel treatments that are easily accessible and less harmful to the general population in order to tackle these helminthiases.

The use of medicinal plants, a major source of molecules with therapeutic properties as far as diseases are concerned due to the existence of natural chemicals, is one way to overcome this resistance issue [9]. Due to the presence of phytochemical elements, medicinal plants are helpful in treating human ailments and play a significant part in healing [10].

*Khaya anthotheca* and *F. albida are* plants used in the center, west, and south of Chad for the treatment of helminthiasis by the local population. Studies have shown that aqueous and organic extracts from the bark of *F. albida* have antimicrobial activities [11]. In the Republic of Korea, the work of Lee et al. [12] showed that *K. anthotheca* has antiplasmodial activity. In order to support the usage of *K. anthotheca* and *F. albida* in the traditional Chadian pharmacopoeia for the treatment of helminthiasis, the present research was carried out to evaluate the anthelmintic activity of these two plants.

## 2. Materials and Methods

### 2.1. Collection and Identification of Plant Materials

Stem barks of *K. anthotheca* and *F. albida* were harvested from the Chadian central region. The plants were cut into small pieces, transported in plastic bags, and identified at the National Plant Identification Centre as *K. anthotheca* (4230/HNC) and *F. albida* (45506/HCN). The plant materials were dried and then grounded into powder and stored at room temperature.

### 2.2. Preparation of Extracts

We used ethanol to make the extraction because during the survey the traditional practitioner told us to use fermented palm wine (ethanol) or infusion to prepare this remedy. Plant extracts were prepared according to the method described by Abdel Azizi et al. [13]. In brief, for the ethanolic extract, 100 g of powder from *K. anthotheca* and *F. albida* were each introduced in 1 L of ethanol and the mixture was stirred for 72 h. The homogenate was filtered using Whatman paper no. 3. The obtained filtrate was dried at 45°C in an oven to get the extract. For the aqueous extract, 100 g of powder was introduced in 1 L of boiled water at 100°C and sealed until cooling. The homogenate was filtered using Whatman paper. The obtained filtrate was dried at 40°C.

#### 2.2.1. Reference Substances

Albendazole and levamisole were used as the positive control while distilled water and DMSO were used as the negative control as described by Cédric et al. [14].

### 2.3. Isolation and Concentration of Embryonated Eggs of *H. polygyrus bakeri*

The floatation technique described by Cédric et al. [8] was used to concentrate fresh eggs. In brief, the parasite eggs floated to the top when two grams of fresh infected stool sample were mixed with a saturated NaCl solution. With the aid of a glass slide, the eggs were removed from the surface. The slides were washed using distilled water to collect the eggs. The suspension of eggs was centrifuged three times at 1500 rpm for 10 min in order to rid of the salt solution from the eggs. These fresh eggs were incubated for 24 h at room temperature to produce embryonated eggs.

### 2.4. Culture and Collection of *H. polygyrus bakeri* Larvae

The technique described by Johnston et al. [15] was used to culture the larvae. Infected feces were mixed with charcoal in the ratio of 1 : 1 until a correct consistency was obtained. The culture was placed on Petri dishes having wet filter paper on their surface and incubated at 27°C for 48 h (L1 larvae), 96 h (L2 larvae), and 7 days for L3 larvae. Water was added to the cultures regularly to prevent the mixture from getting too dry, approximately every 1-2 days.

### 2.5. *Caenorhabditis elegans* Culture

Using the method outlined by Liu et al. [16], *Caenorhabditis elegans* (wild-type Bristol) was cultured. *C. elegans* was cultured on nematode growth media with OP50 (bacteria) as their food source. Synchronized colonies of eggs were incubated at 20°C and hatched in S basalt solution. L1 larvae were then placed on nematode growth medium (NGM) and incubated for a further 36 h to obtain the L4 larvae. The anthelminthic test was performed using these L4 larvae.

### 2.6. *In Vitro* Anthelminthic Activity against *H. polygyrus bakeri* and L4 Larvae of *C. elegans*

The ovicidal and larvicidal activity was assessed using a Worm Microtracker as described by Cédric et al. [14]. In a 96-microplate assay, 50 embryonated eggs were exposed to the extract at different concentrations ranging from 0.078 to 2.5 mg/mL to test for ovicidal activity. The mobility of the L1 larvae was observed on the plate at 25°C for 24 h in the Worm Microtracker. The same procedure was used to test the larvicidal activity (with 50 larvae per well) against L1 to L3 *H. polygyrus* larvae and L4 *C. elegans* larvae, with the exception that the extract concentrations tested for L4 *C. elegance* larvae ranged from 6.25 to 200 *µ*g/mL, and the plates were incubated in the Worm Microtracker at 20°C for 18 h instead. The setup was repeated three times for each treatment and control for both ovicidal and larvicidal activities in the same conditions. An infrared microbeam, which crosses each microtiter well and scanned more than ten times per second, was used by the Worm Microtracker to measure and record the motility of the worms in each well every thirty minutes. The microbeam was interrupted when a worm moved by. The anthelminthic activity was determined as follows:

(1) $$\begin{array}{c} \%\text{ inhibition}=\frac{\text{mobility activity of control}-\text{mobility activity of the test sample}}{\text{mobility activity of control}}\times 100. \end{array}$$

### 2.7. Molecular Docking

**Figure 1:**
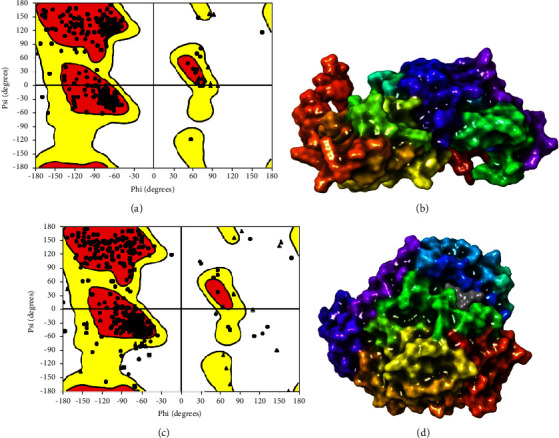
(a) Ramachandran plot of tubulin alpha-1B chain, (b) optimized tubulin alpha-1B chain with the optimized active site (surface mode) [19], (c) Ramachandran plot of tubulin beta chain, and (d) optimized tubulin beta chain with the optimized active site (surface mode) [19].

Docking is the term used to describe the process of predicting the conformation and orientation of a ligand in a particular binding site. In this study, tubulin alpha-1B chain and tubulin beta chain was/were selected as the main target for several anthelmintic substances, based on information from various articles on anthelmintic receptor proteins [17, 18]. The structure of the *C. elegans* tubulin was downloaded from the Protein Data Bank portal (PDB ID: 6E88) as seen in Figures 1(a) and 1(b). A crystal structure of beta tubulin (PDB ID: 1SA0 (tubulin crystals complexed with colchicine binding site))[20–22] from the PDB (https://www.rcsb.org/pdb) was selected and edited after removing the heteroatoms. The merging of nonpolar hydrogens generated polar hydrogens and Gasteiger charges (Figures 1(c) and 1(d)). By considering the relevant ionization states for both acidic and basic amino acid residues, hydrogen bonds associated with pH 7.4 were added, while crystallographic water molecules that lacked 3H bonds were deleted. Shivakumar et al. [23] used the OPLS_2005 force field for the purpose of energy minimization of the crystal structure and we use the OPLS to minimize. Glide and SiteMap applications of Schrodinger suits [19, 24] were used to determine the protein site, and docking was conducted with ligands of *Khaya anthotheca* [25, 26] and *Faidherbia albida* [27–29].

The tubulin alpha-1B chain and tubulin beta chain grid box were created using Maestro's Glide program in the receptor grid generation section. The SiteMap app from Maestro [24] is used for foreseeing receptor active sites. A grid center of the tubulin alpha-1B chain was calculated (X: 240.06, Y: 91.29, Z: 118.29) by creating two boxes with ranges 15 × 15 × 15 and 20 × 20 × 20, which represent the active site of the tubulin alpha-1B chain. The grid center of the tubulin beta chain was calculated (X: 117.09, Y: 89.98, Z: 6.33) by creating two boxes with ranges 20 × 20 × 20 and 30 × 30 × 30, which represent the active site of the tubulin beta chain.

Docking studies were carried out using the Glide module of Schrodinger Maestro software [19]. Possible adduct structures generated by molecular docking were ranked and grouped using the software's score function [30] (Tables 1 and 2). The three-dimensional structure of any complex can be predicted based on the binding properties of the ligand and he target. The “protein preparation wizard” was used to preprocess the protein structure in Maestro [19]. The protein molecule's missing site was filled with hydrogen atoms and crucial bonds using modules' automated state generation and refinement phases. Receptor grid generation was carried out after the optimization process, and the docking scores were examined using different docked ligand conformations [31, 32].

### 2.8. *In Silico* ADME Evaluation

The ADME (absorption, distribution, metabolism, and excretion) properties of *K. anthotheca* and *F. albida* compounds were predicted using QikProp application in Maestro software of Schrodinger suites [33]. The following were the standard parameters for this rule:

1. Molecular weight must not exceed 500
2. Donor that forms hydrogen bond (range permitted: ≤5)
3. Acceptable range for hydrogen bond acceptor is 10 or less
4. LogP expresses high lipophilicity with an acceptable range of ≤5
5. Range of acceptable molar refractivity is 40–130

To determine the ADME properties of these constituents, the QikProp module of Schrödinger Maestro (v12.5) was used [34].

### 2.9. Statistical Analysis

The activity measurements were used to calculate the percentage inhibition using Microsoft Excel. The half maximum inhibitory concentrations (IC_50_) were then determined using the concentration-response curves produced by plotting the logarithm of the concentration as a function of the % inhibition using the GraphPad Prism version 8 software. The Glide module of the Schrodinger Maestro software [19] was used to conduct the docking studies. The software's score function was used to classify and rank various potential adduct structures produced by molecular docking.

## 3. Results

### 3.1. Anthelminthic Activity against *H. polygyrus bakeri*

Table 3 presents the percentage (%) inhibition of hatching, larvae motility, and half maximal inhibitory concentration (IC_50_) of *K. anthotheca* and *F. albida* extracts. The IC_50s_ of the aqueous extracts of the two plants (IC_50_: 0.6212 mg/mL and 0.71 mg/mL, respectively) were comparable on egg hatching of *H. polygyrus* with no significant difference (*p* ≥ 0.05) with respect to the ethanol extracts (IC_50_: 0.70 mg/mL and 0.81 mg/mL, respectively). The aqueous and ethanolic extracts of *F. albida* at a concentration of 2.5 mg/mL showed the same activity as albendazole (100 ± 0.00). The aqueous and ethanolic extracts of *K. anthotheca* and *F. albida* showed a slightly lower inhibitory activity than the positive control (albendazole) on the L1 larvae with a percentage inhibition of 99.29 ± 0.25; 99.29 ± 0.35 (*K. anthotheca*); 100 ± 0.00; and 99.8 ± 0.12 (*F. albida*) at a concentration of 2.5 mg/mL. There is no significant difference between the percentage inhibition of the extracts and albendazole (*p* < 0.05) on the L1 larvae. Aqueous extracts act more effectively than ethanolic extracts with an IC_50_ of 0.5588 and ∼9.858*e* − 005 mg/mL, respectively, for *K. anthotheca* and *F. albida* on the L1 larvae.

Moreover, at the concentration of 2.5 mg/ml, the aqueous and ethanolic extracts have an inhibition percentage of 98.50 ± 0.59 and 97.50 ± 0.41 for *K. anthotheca* and 99.68 ± 0.04 and 95.65 ± 1.12 for *F. albida* on the L2 larvae and the two plants show similar activity to albendazole. There is no significant difference between the percentage's inhibition of the extracts at the concentration of 2.5 mg/mL and albendazole for the L2 larvae. For the L2 stage larvae, aqueous extracts from both *K*. *anthotheca* (IC_50_ 0.237 mg/mL) and *F. albida* (0.072 mg/mL) were more effective than the ethanolic extracts (*K. anthotheca*: IC_50_ 0.21 mg/mL and *F. albida* 0.12 mg/mL). The aqueous and ethanol extracts of *K. anthotheca* have a respective IC_50_ of 0.02 and 0.06 mg/mL on L3 larvae which are more active than the aqueous and ethanol extracts of *F. albida* with a respective IC_50_ of 0.11 and 0.31 mg/mL.

### 3.2. Effect of Extracts on L4 Larvae of *C. elegans*

Table 1 shows the percentage mortality vs concentration of extracts (mg/ml) for L4 larvae of *C. elegans*. Aqueous extracts of *K. anthotheca* (IC_50_ of 0.2775 *µ*g/mL) and *F. albida* (0.5115 *µ*g/mL) act more effectively than ethanolic extracts. At the concentration of 2.5 *µ*g/mL, aqueous and ethanolic extracts of *K. anthotheca* (93.13 ± 1.87 and 90.70 ± 2.13) as well as *F. albida* (90.41 ± 0.07 and 72.62 ± 0.17) exhibited activity lower than levamisole (100 ± 0.00). This difference is significant (*p* < 0.05).

### 3.3. *In Silico* Approach and Molecular Docking Analysis of Anthelminthic

A Glide module was used to perform molecular docking between the target protein and ligands [35, 36]. The interaction of some ligands with amino acids in the target protein resulted in a significant docking score. Docking scores for the top 5 ligands are depicted in Tables 2, 4, 5, 6.

Molecular docking methodologies including HTVS, SP, and XP were used to screen the compounds extracted from *K. anthotheca* and *F. albida*. The top 15% of the most stable ligands were evaluated for docking scores at each stage. The most stable ligands were evaluated for XP docking score on their structures.

**Figure 2:**
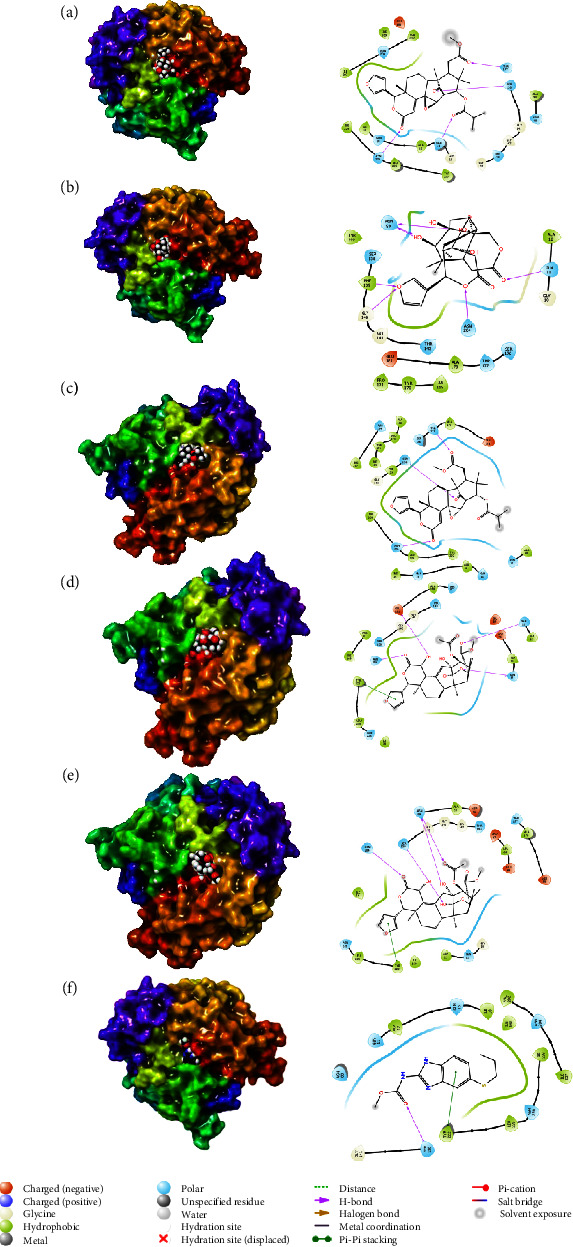
3D and 2D interactions of tubulin alpha-1B chain's protein and ligands of *Khaya anthotheca*: (a) 14,15-didehydroruageanin A, (b) anthothecanolide, (c) 3-O-acetylanthothecanolide, (d) khayalenoid D, (e) khayalenoid C, and (f) albendazole, based on Maestro of Schrödinger suites [19].

The alpha tubulin-1B chain's docking scores and binding interactions with the top five compounds of *K. anthotheca* are compared in Figure 2 and Table 2. 14,15-Didehydroruageanin A is the most active compound in *K. anthotheca*, due to its abundant hydroxyl functional groups, carboxylic acid groups, and aromatic rings, which enable it to have a stronger inhibitory effect on receptor proteins.

Due to its hydroxyl, ether, and carbonyl groups, anthothecanolide's composition also exhibits good inhibitory activity against asparagine, tyrosine, phenylalanine, and glutamic acid.

As with the preceding compounds that contained asparagine, serine, and threonine of the backbone, 3-O-acetylanthothecanolide is connected to the receptor through its carbonyl groups. In addition to its oxygen and hydroxyl groups, the khayalenoid D compound also binds to amino acids such as asparagine, glycine, and phenylalanine, showing its inhibitory effect.

The fifth compound, khayalenoid C, shows the same inhibitory effect on the receptor protein due to its oxygen atoms as carbonyl groups and hydroxyl groups and binding to asparagine, tyrosine, and serine.

**Figure 3:**
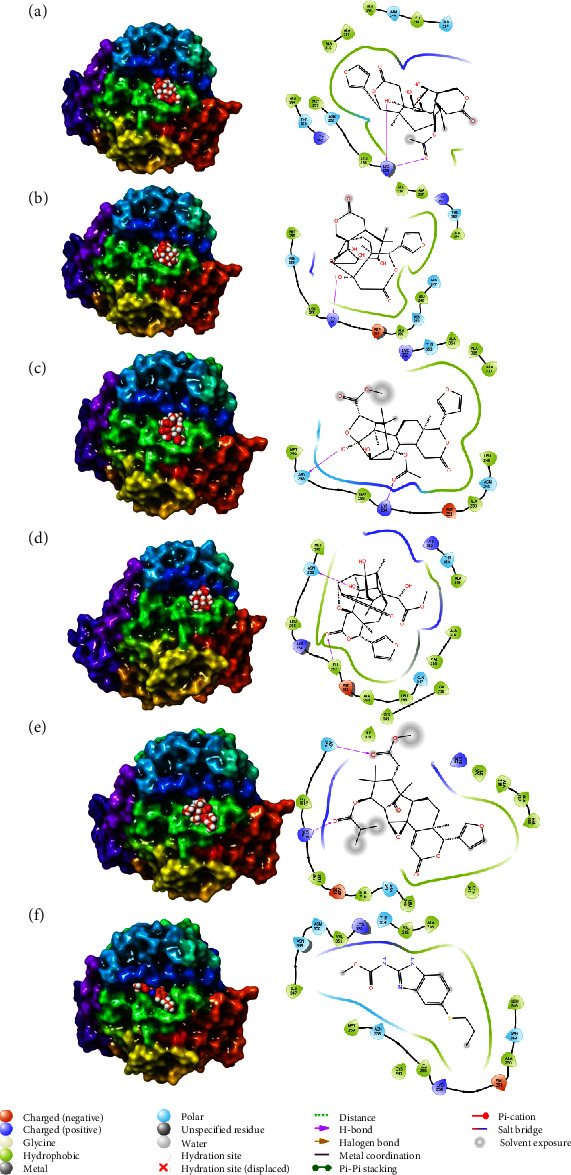
3D and 2D interactions of tubulin beta chain's protein and ligands of *Khaya anthotheca*: (a) [(1R,6S,11S,16S,19R)-6-(furan-3-yl)-2,19,20-trihydroxy-7,17,17-trimethyl-4,14-dioxo-5,13,21-trioxahexacyclo[17.2.1.01,10.02,7.011,16.011,20]docosan-18-yl] acetate, (b) (1R,6S,11S,16S,19R)-6-(furan-3-yl)-2,18,19,20-tetrahydroxy-7,17,17-trimethyl-5,13,21-trioxahexacyclo[17.2.1.01,10.02,7.011,16.011,20]docosane-4,14-dione, (c) methyl (1S,2R,3S,5S,6S,8S,9R,16R,17R)-8-acetyloxy-16-(furan-3-yl)-3,9-dihydroxy-2,7,7,17-tetramethyl-14-oxo-4,15-dioxapentacyclo[9.8.0.02,6.03,9.012,17]nonadec-11-ene-5-carboxylate, (d) 1-O-deacetylkhayanolide E, (e) [(1S,2R,9R,10R,13R,14R,15S,17R)-9-(furan-3-yl)-15-(2-methoxy-2-oxoethyl)-10,14,16,16-tetramethyl-7,18-dioxo-3,8-dioxapentacyclo[12.3.1.02,4.04,13.05,10]octadec-5-en-17-yl] 2-methylpropanoate, and (f) albendazole, based on Maestro of Schrödinger suites [19].

Docking scores and binding interactions of the tubulin beta chain with the top five compounds of *K. anthotheca* have been compared in Figure 3 and Table 4.

*K. Anthotheca* and the first compound ([(1R,6S,11S,16S,19R)-6-(furan-3-yl)-2,19,20-trihydroxy-7,17,17-trimethyl-4,14-dioxo-5,13,21-trioxahexacyclo [17.2.1.01,10.02,7.011,16.011,20]docosan-18-yl] acetate), show the greatest amount of interactions due to the interaction between the amino acid lysine with the hydroxyl groups and carbonyl groups. Most of the interactions between tubulin beta chain and 1,6-(furan-3-yl)-2,18,19,20-tetrahydro-7,17,17-trimethyl-5,13,21-trioxahexacyclo[17.2.1.01,10.02,7.011,16.011,20]docosane-4,14-dione are due to the interaction between lysine amino acid and the hydrogen of hydroxyl group.

Because of the carbonyl and hydroxyl groups in the backbone, methyl (1S,2R,3S,5S,6S,8S,9R,16R,17R)-8-acetyloxy-16-(furan-3-yl)-3,9-dihydroxy-2,7,7,17-tetramethyl-14-oxo-4,15-dioxapentacyclo[9.8.0.02,6.03,9.012,17]nonadec-11-ene-5 carboxylate interacts with lysine and asparagine. The interactions between the 1-O-deacetylkhayanolide E structure and Asn and Leu are caused by hydroxyl and carbonyl groups.

Interactions of [(1S,2R,9R,10R,13R,14R,15S,17R)-9-(furan-3-yl)-15-(2-methoxy-2-oxoethyl)-10,14,16,16-tetramethyl-7,18-dioxo-3,8-dioxapentacyclo[12.3.1.02,4.04,13.05,10] octadec-5-en-17-yl] 2-methylpropanoate is based on carbonyl groups and hydrogen donors to amino acids of lysine and asparagine. Compared to albendazole, which has a carbonyl group and has an imine and imide pharmacophore on its structure, it shows similar interactions with the tubulin beta chain.

**Figure 4:**
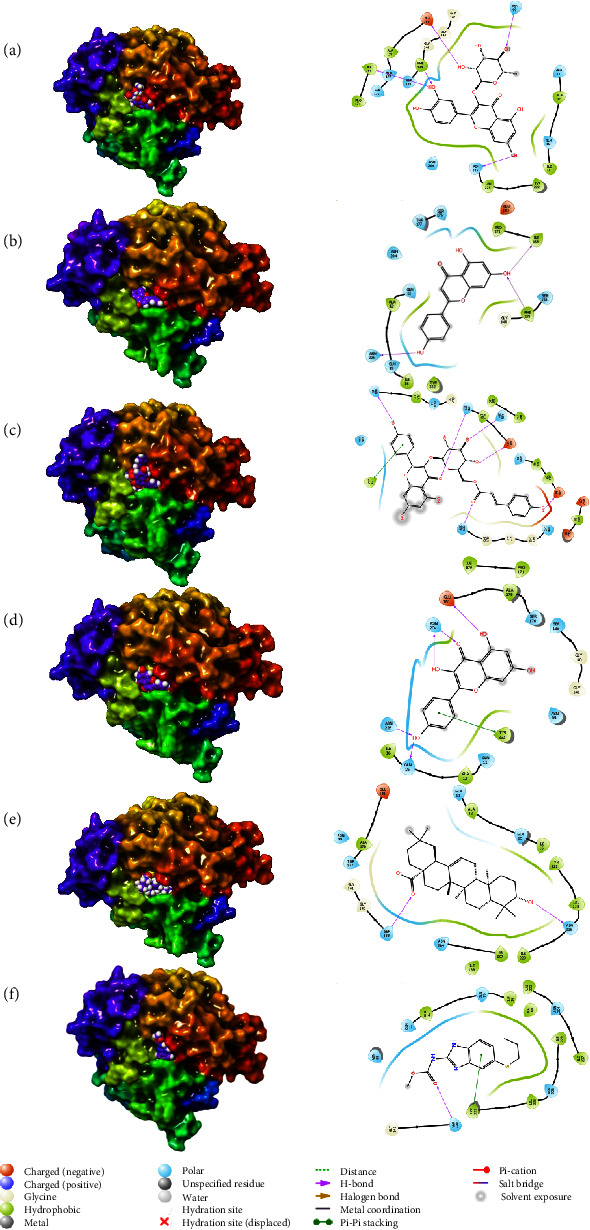
3D and 2D interactions of tubulin alpha-1B chain's protein and ligands of *Faidherbia albida*: (a) quercitrin, (b) apigenin, (c) tiliroside, (d) kaempferol, (e) oleanic acid, and (f) albendazole, based on Maestro of Schrödinger suites [19].

The alpha tubulin-1B chain's docking scores and binding interactions with the top five compounds are compared in Figure 4 and Table 5. In *F. albida*, quercitrin has the most interactions with amino acids asparagine, phenylalanine, glutamic acid, and also isoleucine, due to its abundant hydroxyl functional groups, which makes it have a significant inhibitory effect on the receptor protein. The apigenin compound shows a good inhibitory effect due to its hydroxyl groups and interaction with amino acids such as asparagine, phenylalanine, and isoleucine.

Tiliroside compound is attached to the receptor due to its abundant hydroxyl groups and aromatic ring, such as the previous compounds with asparagine, glycine, tyrosine, and glutamic acid.

The composition of kaempferol is no exception to this rule and shows its inhibitory effect due to its flavonoid backbone, hydroxyl and carbonyl groups, and binding to thyrosine, glutamine, glutamic acid, and asparagine amino acids. The fifth compound of *F. albida*, oleanic acid also shows its inhibitory effect on the receptor protein due to its hydroxyl and carbonyl groups and binding to asparagine, and serine amino acids.

Table 6 compares the tubulin beta chain's docking scores and binding interactions with the top five compounds of *F. albida*. First, tiliroside interacts with tubulin beta chain receptors. A hydrogen donor is involved in these interactions because it provides hydrogen to the receptor's valine and alanine. The hydroxyl groups of apigenin interact with the valine and asparagine of the receptor, as well as the first structure. Kaempferol, a flavonoid, provides hydrogen to the receptor's valine amino acid. The interactions between betulin and receptors are caused by the hydroxyl groups in this compound. Despite lupeol's differences, it has the same interactions with receptors since it provides hydrogen to Valine amino acids.

As a control, albendazole exhibits similar interactions with the tubulin beta chain due to imine and imide pharmacophores on its structure.

### 3.4. Evaluating the ADME

The drug-like behavior of a chemical agent is evaluated by the ADME property. Tables 2, 4, 5, 6 show that the constituents have good pharmacokinetic profiles and are not demonstrated in violation of the Lipinski rule by the constituents. Also, they do not have any mutagenic or carcinogenic properties and are nonhazardous.

## 4. Discussion

The aqueous and ethanolic extracts of *K. anthotheca* and *F. albida* showed good ovicidal activity against *H. polygyrus.* Aqueous extracts of *K. anthotheca* and *F. albida* were more active than ethanolic extracts. These observations are contrary to those observed by Wabo et al. [37] and Cédric et al. [8] on *H. polygyrus bakeri* eggs. The fact that egg hatching did not occur could be justified by the active substances in the extract which crossed the egg membranes, reached the larvae, and penetrated their cuticle, thus causing their death. Moreover, when larvae seek to absorb water from the surrounding environment, they swell and break the membrane to escape, and when they find that the latter contains molecules harmful to its survival, it then prefers to stay and die in the membrane/shell.

Regarding larvicidal activity, aqueous and ethanolic extracts of *K*. *anthotheca* and *F. albida* showed significant inhibitory activity with increasing concentration. These observations were similar to those obtained by Payne et al. [38]. This could be explained by the active ingredients contained in the extracts blocking the receptors, thus paralyzing the larva. Lem et al. [39] mentioned that active compounds present in foods can pass through the intestinal wall of larvae and access the body's circulatory system. In addition, active compounds such as tannin can bind to the nematode cuticle, destabilize the membrane, and increase cell permeability by combining with sterols, resulting in death [8].

Aqueous and ethanolic extracts of *K. anthotheca* exhibit larvicidal activity against L3 larvae of *H. Polygyrus bakeri.* These results show that both extracts of this plant are active. These observations were similar to those obtained by Ademola et al. [40], who found a good inhibition with the extract of *K. senegalensis* and those of Kolapo et al. [41] with *K. grandifoliola.* This activity is justified by the presence of bioactive compounds such as terpenes, alkaloids, flavonoids, phenols, and amthamines, which could contain anthelmintic properties. As for *F. albida*, the anthelmintic activity of the aqueous and ethanolic extracts was effective against L4 larvae of *C. elegans.* These observations validate those obtained by Mamat et al. [42], who also found the good activity of *F. albida* extracts against *C. elegans.* This activity could be justified by the presence of the active principle contained in the plants and polyphenolic compounds which could have anthelmintic properties.

*In silico* studies and ADME assessments have revealed that kaempferol and apigenin exhibit docking scores comparable to albendazole against *α*- and *β*-tubulin, indicating strong interactions. The hydroxyl and carbonyl groups, along with the flavonoid backbone of these compounds, facilitate effective binding to tubulin chains. These findings support the potential of Kaempferol and Apigenin as anthelmintic agents.

Several tubulin-binding herbicides exhibit activity against various protozoal parasites, and cell-based studies have shown that, similar to their effects in plants, the target in these protozoa is tubulin [43]. Tubulin is an antiprotozoal drug target. The benzimidazole class of anthelmintics, including albendazole, thiabendazole, mebendazole, and fenbendazole are broad-spectrum agents effective against gastrointestinal nematodes and, at higher concentrations, some trematodes. These anthelmintics function by binding to *β*-tubulin. *β*-tubulin, along with *α*-tubulin, polymerizes to form microtubules, which are essential structures within the cells of both nematodes and their host organisms [44]. The mechanism of action of benzimidazoles became clearer when mebendazole was observed to cause damage to the intestinal cells of Ascaris [45]. It was found that this damage resulted from the loss of cytoplasmic tubules in the intestinal cells and the teguments of cestodes and nematodes [46]. This disruption was associated with impaired transport of secretory vesicles and an inability of the intestinal cells to absorb glucose, ultimately leading to parasite starvation. In ascaris, mebendazole was found to bind to cytoplasmic proteins with molecular weights of 50 kDa and 100 kDa, corresponding to tubulin monomers and dimers. Benzimidazole anthelmintics were shown to compete for the *β*-tubulin-binding site with colchicine, a known inhibitor of cell division during metaphase [47]. Microtubules are crucial for various intracellular functions, including the transport of cytoplasmic secretory vesicles [48].

## 5. Conclusion

The aqueous extracts of *K. anthotheca* and *F. albida* showed more active anthelmintic activity *in vitro* than the ethanolic extracts on the eggs, L1, L2, and L3 of *H. polygyrus bakeri* and L4 larvae of *C. elegans. In silico* studies and ADME assessments have revealed that kaempferol and apigenin exhibit docking scores comparable to albendazole against *α*- and *β*-tubulin, indicating strong interactions. However, further *in vivo* anthelminthic and toxicity tests are required to validate their ethnobotanical usage by the local population.

### 5.1. Limitation of the Present Findings

We were unable to identify the different ligands that might have been present in our extract by performing an HPLC fingerprint on the extracts due to our limited resources.

## Figures and Tables

**Table 1:** Percentage mortality vs concentration of extracts (mg/mL) for L4 larvae of *C. elegance*.

Plants	Concentrations (*µ*g/mL)									
	Extracts	3.125	6.25	12.5	25	50	100	IC_50_	Levamisole (5 nM)	Negative control
*K. anthotheca*	Aqueous	30.39 ± 0.07^a^	44.45 ± 0.19^b^	48.13 ± 0.65^c^	59.25 ± 0.28^d^	69.50 ± 0.10^e^	93.13 ± 1.87^f^	**1.89**	100.0 ± 0.00^g^	0.00 ± 0.00
	Ethanol	15.73 ± 0.49^a^	28.29 ± 1.63^b^	31.55 ± 0.12^c^	46.43 ± 1.24^d^	66.20 ± 0.45^e^	90.70 ± 2.13^f^	**2.05**	100.0 ± 0.00^g^	00.0 ± 0.00

*F. albida*	Aqueous	15.49 ± 0.09^a^	37.5 ± 0.14^b^	40.39 ± 0.18^c^	60.38 ± 1.19^d^	86.33 ± 0.08^e^	90.51 ± 0.07^f^	**0.51**	100.0 ± 0.00^g^	00.0 ± 0.00
	Ethanol	16.21 ± 0.65^a^	24.59 ± 1.14^b^	28.58 ± 0.15^c^	40.15 ± 0.00^d^	58.09 ± 0.81^e^	72.64 ± 0.17^f^	**1.57**	100.0 ± 0.00^g^	00.0 ± 0.00

The results are the mean ± SD of triplicate tests evaluated. a, b, c, d, e, f, and g values with the same superscript letter in the same column are not significant with different at *P* ≥ 0.05. The bold values indicates the IC_50_.

**Table 2:** Assessment of ADME and docking scores conducted for ligands of *Khaya anthotheca* and tubulin alpha-1B chain.

No.	Compounds	Solute molecular weight^1^	QPlogHERG^2^	QPPCaco^3^ (nm/s)	QPPMDCK^4^ (nm/s)	Rule of five^5^	Rule of three^6^	Docking score
1	14,15-Didehydroruageanin A	554.6	−5.159	318.699	143.735	1	0	−6.648
2	Anthothecanolide	504.5	−3.492	69.06	27.523	1	1	−6.455
3	3-O-Acetylanthothecanolide	546.6	−4.106	75.845	30.457	2	0	−5.938
4	Khayalenoid D	560.6	−4.316	90.883	37.033	2	1	−5.818
5	Khayalenoid C	560.6	−4.438	74.771	29.991	2	1	−5.253
6	Albendazole (positive control)	265.3	−5.189	607.708	439.375	0	0	−4.013

^1^130–725; ^2^concern below −5; ^3^*a* < 25, poor, and *a* > 500, great; ^4^*a* < 25, poor, and *a* > 500, great; ^5^maximum is 4; ^6^maximum is 3.

**Table 3:** Percentage (%) inhibition of hatching, larvae motility, and half maximal inhibitory concentration (IC_50_).

Anthelminthic test	Plants	Concentrations (mg/mL)									
		Extracts	0.078	0.151	0.315	0.625	1.25	2.5	IC_50_	Albendazole (5 *µ*g/ml)	Negative control
% inhibition of hatching	*K. anthotheca*	Aqueous	46.15 ±0 .34^a^	46.63 ± 0.04^a^	59.40 ± 1.74^b^	78.69 ± 0.036^c^	88.24 ± 0.53^d^	99.24 ± 0.48^e^	**0.62**	100.0 ± 0.00^e^	0.00 ± 0.00
		Ethanol	48.58 ± 0.04^a^	59.93 ± 1.74^b^	66.57 ± 0.03^c^	74.68 ± 0.53^d^	91.40 ± 0.48^e^	100.0 ± 0.00^f^	**0.70**	100.0 ± 0.00^f^	0.00 ± 0.00
	*F. albida*	Aqueous	58.84 ± 1.07^a^	64.85 ± 1.18^b^	76.51 ± 0.92^c^	85.78 ± 0.72^d^	97.99 ± 0.63^e^	100.0 ± 0.00^e^	**0.71**	100.0 ± 0.00^e^	0.00 ± 0.00
		Ethanol	58.13 ± 0.81^a^	60.64 ± 0.41^b^	72.48 ± 0.54^b^	83.42 ± 1.18^c^	99.00 ± 0.63^d^	100.0 ± 0.0^d^	**0.81**	100.0 ± 0.00^d^	0.00 ± 0.00

% inhibition of L1 larvae motility	*K. anthotheca*	Aqueous	33.67 ± 1.008^a^	35.17 ± 1.14^a^	62.69 ± 1.28^b^	70.90 ± 0.16^c^	93.18 ± 0.66^d^	99.29 ± 0.25^e^	**0.44**	100.0 ± 0.00^e^	0.00 ± 0.00
		Ethanol	27.55 ± 0.83^a^	44.02 ± 1.25^b^	56.21 ± 1.08^c^	64.65 ± 1.46^d^	91.78 ± 1.008^e^	99.29 ± 0.35^f^	**0.56**	100.0 ± 0.00^f^	0.00 ± 0.00
	*F. albida*	Aqueous	7.430 ± 0.03^a^	61.11 ± 0.40^b^	70.58 ± 1.03^c^	76.92 ± 0.59^d^	98.72 ± 0.008^e^	100 ± 0.0^e^	**0.10**	100.0 ± 0.00^e^	0.00 ± 0.00
		Ethanol	12.10 ± 0.80^a^	17.77 ± 0.10^b^	30.44 ± 1.49^c^	62.01 ± 0.07^d^	86.63 ± 0.70^e^	99.8 ± 0.12^f^	**0.75**	100.0 ± 0.00^f^	0.00 ± 0.00

% inhibition of L2 larvae motility	*K. anthotheca*	Aqueous	79.07 ± 0.12^a^	83.76 ± 0.59^b^	88.62 ± 0.47^c^	92.81 ± 0.84^d^	97.07 ± 0.42^e^	98.60 ± 0.59^e^	**0.24**	100.0 ± 0.00^e^	0.00 ± 0.00
		Ethanol	54.01 ± 1.15^a^	62.63 ± 0.43^b^	77.00 ± 0.38^c^	85.37 ± 1.57^d^	94.08 ± 0.39^e^	97.60 ± 0.41^f^	**0.21**	100.0 ± 0.00^f^	0.00 ± 0.00
	*F. albida*	Aqueous	82.28 ± 0.32^a^	86.92 ± 1.58^b^	90.74 ± 0.12^b^	93.90 ± 0.30^c^	98.15 ± 0.16^c^	99.68 ± 0.04^d^	**0.07**	100.0 ± 0.00^d^	0.00 ± 0.00
		Ethanol	65.58 ± 1.244^a^	76.03 ± 1.00^b^	83.19 ± 0.75^b^	85.62 ± 1.75^b^	91.99 ± 0.16^c^	95.65 ± 1.16^c^	**0.12**	100.0 ± 0.00^c^	0.00 ± 0.00

% inhibition of L3 larvae motility	*K. anthotheca*	Aqueous	9.76 ± 1.44^a^	51.75 ± 0.71^b^	66.49 ± 1.70^c^	74.87 ± 1.08^d^	84.92 ± 1.08^e^	92.63 ± 0.62^ef^	**0.02**	100.0 ± 0.00^f^	0.00 ± 0.00
		Ethanol	7.26 ± 0.83^a^	27.67 ± 0.41^b^	33.65 ± 0.82^b^	53.09 ± 0.85^c^	67.16 ± 1.18^d^	86.18 ± 5.82^e^	**0.63**	100.0 ± 0.00^f^	0.00 ± 0.00
	*F. albida*	Aqueous	5.75 ± 0.25^a^	36.16 ± 1.02^b^	54.76 ± 0.82^c^	66.49 ± 1.31^d^	82.24 ± 0.62^e^	91.88 ± 0.78^f^	**0.11**	100.0 ± 0.00^g^	0.00 ± 0.00
		Ethanol	5.25 ± 0.43^a^	31.64 ± 0.41^b^	46.22 ± 1.43^c^	55.77 ± 1.23^d^	81.40 ± 0.20^e^	91.37 ± 0.8^f^	**0.31**	100.0 ± 0.00^g^	0.00 ± 0.00

The results are the mean ± SD of triplicate tests evaluated. a, b, c, d, e, f, and g values with the same superscript letter in the same column are not significantly different at *p* ≥ 0.05. The bold values indicates the IC_50_.

**Table 4:** Assessment of ADME and docking scores conducted for ligands of *Khaya anthotheca* and tubulin beta chain.

No.	Compounds	Solute molecular weight^1^	QPlogHERG^2^	QPPCaco^3^ (nm/s)	QPPMDCK^4^ (nm/s)	Rule of five^5^	Rule of three^6^	Docking score
1	[(1R,6S,11S,16S,19R)-6-(furan-3-yl)-2,19,20-trihydroxy-7,17,17-trimethyl-4,14-dioxo-5,13,21-trioxahexacyclo[17.2.1.01,10.02,7.011,16.011,20]docosan-18-yl] acetate	546.21	−3.337	137.397	57.89	2	0	−5.611
2	(1R,6S,11S,16S,19R)-6-(furan-3-yl)-2,18,19,20-tetrahydroxy-7,17,17-trimethyl-5,13,21-trioxahexacyclo[17.2.1.01,10.02,7.011,16.011,20]docosane-4,14-dione	504.2	−2.896	88.723	36.083	1	1	−5.424
3	Methyl (1S,2R,3S,5S,6S,8S,9R,16R,17R)-8-acetyloxy-16-(furan-3-yl)-3,9-dihydroxy-2,7,7,17-tetramethyl-14-oxo-4,15-dioxapentacyclo[9.8.0.02,6.03,9.012,17]nonadec-11-ene-5-carboxylate	544.231	−3.592	200.129	86.926	1	1	−5.348
4	1-O-Deacetylkhayanolide E	516.2	−3.696	76.49	30.737	1	1	−5.07
5	[(1S,2R,9R,10R,13R,14R,15S,17R)-9-(furan-3-yl)-15-(2-methoxy-2-oxoethyl)-10,14,16,16-tetramethyl-7,18-dioxo-3,8-dioxapentacyclo[12.3.1.02,4.04,13.05,10]octadec-5-en-17-yl] 2-methylpropanoate	554.252	−4.822	288.406	129.027	1	0	−4.986
6	Albendazole (positive control)	265.34	−5.136	611.836	440.617	0	0	−3.911

^1^130–725; ^2^concern below −5; ^3^*a* < 25, poor, and *a* > 500, great; ^4^*a* < 25, poor, and *a* > 500, great; ^5^maximum is 4; ^6^maximum is 3.

**Table 5:** Assessment of ADME and docking scores conducted for ligands of *Faidherbia albida* and Tubulin alpha-1B chain.

No.	Compounds	Solute molecular weight^1^	QPlogHERG^2^	QPPCaco^3^ (nm/s)	QPPMDCK^4^ (nm/s)	Rule of five^5^	Rule of three^6^	Docking score
1	Quercitrin	448.4	−4.955	5.984	1.957	2	2	−7.982
2	Apigenin	270.24	−5.114	114.491	47.533	0	0	−7.662
3	Tiliroside	594.5	−5.989	1.48	0.432	3	2	−7.444
4	Kaempferol	286.24	−5.201	51.24	19.934	0	0	−7.325
5	Oleanic acid	456.7	−1.788	327.01	187.969	1	1	−5.964
6	Albendazole (positive control)	265.3	−5.189	607.708	439.375	0	0	−4.013

^1^130–725; ^2^concern below −5; ^3^*a* < 25, poor, and *a* > 500, great; ^4^*a* < 25, poor, and *a* > 500, great; ^5^maximum is 4; ^6^maximum is 3.

**Table 6:** Assessment of ADME and docking scores conducted for ligands of *Faidherbia albida* and tubulin beta chain.

No.	Compounds	Solute molecular weight^1^	QPlogHERG^2^	QPPCaco^3^ (nm/s)	QPPMDCK^4^ (nm/s)	Rule of five^5^	Rule of three^6^	Docking score
1	Tiliroside	594.137	−6.185	3.801	1.198	3	2	−8.514
2	Apigenin	270.053	−3.888	1752.501	907.227	1	1	−6.85
3	Kaempferol	286.048	−5.093	56.89	22.32	0	0	−6.54
4	Betulin	442.381	−5.182	117.919	49.073	0	0	−5.47
5	Lupeol	426.386	−3.788	4457.969	2488.802	1	1	−4.856
6	Albendazole (positive control)	265.34	−5.136	611.836	440.617	0	0	−3.911

^1^130–725; ^2^concern below −5; ^3^*a* < 25, poor, and *a* > 500, great; ^4^*a* < 25, poor, and *a* > 500, great; ^5^maximum is 4; ^6^maximum is 3.

## Data Availability

All data generated and analysed are included in this research article.
